# Optimal neural coding in networks of heterogeneous neurons

**DOI:** 10.1186/1471-2202-13-S1-P96

**Published:** 2012-07-16

**Authors:** Jorge F Mejias, Andre Longtin

**Affiliations:** 1 Department of Physics, University of Ottawa, Ottawa, K1N 6N5 Ontario, Canada; 2 Centre for Neural Dynamics, University of Ottawa, Ottawa, K1N 6N5 Ontario, Canada

## 

Neural systems display a prominent level of heterogeneity in synaptic, neuron, and network properties. Even among same-class neurons, differences in excitability properties, for instance, may be present in the system, and this could have strong implications for information processing. A well-known example is the so called *population coding*, a strategy which relies on the particular responses of individual neurons of a network to code information. This occurs for instance in V1 cortical networks, where the orientation preference of neurons allows for efficient information coding [1]. However, not many studies have addressed the role of neural heterogeneity in other neural coding strategies. Interestingly, cell-to-cell differences have been recently found to be relevant in synchronization of inhibitory networks [2], coherent activity in electrically coupled neurons [3], synchronized bursting events [4], global detection of weak signals [5], and efficient coding for communication signals [6], highlighting a possible role of heterogeneity on neural information processing. Still, neither the effect of heterogeneity on the dynamics of neural populations nor its influence on neural coding are yet fully understood.

In this work, we present a theoretical and numerical study of how different strategies of neural coding (concretely, rate coding and temporal coding) are affected by the presence of heterogeneity in the properties of the neurons in the network, paying special attention to their ability to process and codify incoming signals. We carry out this study in two different systems: the first one, which is conceptually simpler, is a fully-connected network of excitatory integrate-and-fire neurons with small synaptic delays. The second one is more realistic, and consists in a sparsely connected network of excitatory and inhibitory integrate-and-fire neurons with small synaptic delays. For both systems, a certain level of heterogeneity is introduced in the neuron firing thresholds, that is, each neuron in the network has a particular degree of excitability. Our aim is to investigate the effect of such heterogeneity level in the strategies used by the network to process and codify neural signals.

Our results show that neural heterogeneity has important effects in several properties of interest in neural networks, such as the mean firing rate or the synchronization properties of the network. Then, we find that these effects have strong consequences for two main information processing strategies used in many brain areas: rate coding and temporal coding. More precisely, we show, theoretically and numerically, that certain heterogeneity values- which lie within the physiological rage found in actual neurons- optimize the transmission of information in rate and temporal codes in a stochastic resonance fashion, suggesting that cell-to-cell differences are not useful only for population coding, but also for more general coding strategies. Interestingly, we find that networks of heterogeneous neurons tend to behave differently to input changes (such as increments in input rate) depending on the neural coding strategy they are operating in. Such finding suggests the existence of heterogeneity-induced features which are specific for each neural coding strategy.
